# Social media influencers and followers’ conspicuous consumption: The mediation of fear of missing out and materialism

**DOI:** 10.1016/j.heliyon.2024.e36387

**Published:** 2024-08-15

**Authors:** Thi Cam Tu Dinh, Yoonjae Lee

**Affiliations:** Department of Business Administration, Yeungnam University, Gyeongsan, 38541, South Korea

**Keywords:** Conspicuous consumption, Fear of missing out (FOMO), Desire to mimic, Materialism, Social comparison, Social media influencers (SMIs)

## Abstract

This study addresses the existing gap in theoretical and empirical research concerning the impact of social media influencers (SMIs) on followers' purchasing decisions. The primary aim is to explore and elucidate followers' journey from exposure to SMIs to the manifestation of conspicuous consumption. Grounded in the stimulus-organism-response framework and self-determination theory, the research proposes a dual model focusing on mediating factors such as social comparison, desire to mimic, materialism, and fear of missing out (FOMO). To achieve this objective, a survey targeting 272 respondents was conducted on the MTurk platform. The study findings reveal that exposure to SMIs triggers social comparisons and FOMO, subsequently influencing the acquisition of conspicuous products. Additionally, the study identifies that exposure to SMIs amplifies the desire to mimic and stimulates materialistic tendencies, thereby contributing to conspicuous consumption. The proposed Intrinsic-Extrinsic Consumption Motivation Model emerges as a novel framework to enhance our understanding of how SMIs influence conspicuous consumption, providing valuable insights for developing effective advertising programs.

## 1 Introduction

Social media influencers (SMIs) are the trendsetters in society— “social media stars” [1]—who usually display a noticeably luxurious lifestyle [2]. The emergence of social media influencers (SMIs) is one of the key determinants affecting customer behaviors [3]. SMIs have become successful tools for companies to connect with potential customers [4], and the importance of influencers in marketing activities has increased in recent years [5]. For example, as indicated in a global report from 2024, 85 % of marketers believe that influencer marketing is an effective form of marketing, and 60 % of them intend to increase their influencer marketing budget throughout 2024 [6]. Therefore, SMIs are potential endorsers when they generate a range of buzzwords and create cost-efficient and effective marketing trends [7].

As the influential role of SMIs in shaping consumer behavior becomes increasingly apparent, it is crucial to delve deeper into the mechanisms underlying their impact. SMIs leverage social media platforms through posts, tweets, blogs, and vlogs to actively involve and influence their followers [8]. According to Sekhon, Bickart, Trudel and Fournier [9], products endorsed by SMIs are more trusted and likely to be consumed than those supported by their peers. SMIs have significant power over consumer attitudes and behaviors [10] because followers are likelier to use endorsed products to experience the same value. Therefore, followers idealize SMIs and purchase products endorsed by them to emulate and enhance their own self-status. Recognizing the substantial influence SMIs wield over consumer attitudes and behaviors, it is imperative to underscore the significance of exploring SMIs' impact comprehensively.

Social media users tend to consume products that align with current trends to garner attention, enhance their social standing, and express their self-image [11]. For followers, SMIs serve as ideal models, influencing their choices of conspicuous products to make an impression on others. Although existing research predominantly delves into the impact of SMIs on social media users through factors such as credibility, expertise, attractiveness, and congruity [[12], [11], [13], [14]], there remains a research gap in understanding the sequential progression of consumers' experiences, especially within a comprehensive framework—from exposure to SMIs to the formation of purchasing intentions. This gap underscores the need for further exploration into the specific factors driving the influence exerted by SMIs on their followers [7,15].

To address this, the present study employs the Stimulus-Organism-Response (SOR) framework and Self-Determination Theory (SDT) as background theories to explain followers' behaviors. This study uses the SOR framework as an overarching theory, as previous researchers have demonstrated its predictive power in understanding how retail consumers respond to new environmental stimuli [16]. The SOR framework posits that environmental cues can stimulate self-assessment state, which can lead to specific behavior or response. In this case, SMIs are stimulating cues that can trigger followers' reactions and subsequently lead to their behavior, such as conspicuous consumption [17]. Thus, it's reasonable that there exists a potential intermediary between the exposure to SMIs and the ensuing reactions of followers, which will evoke conspicuous consumption desires. Moreover, while previous research has explored the direct motivations underlying the effect of SMIs on followers [7,14,18], this study proposes a dual framework that better elucidates conspicuous consumption motivation following exposure to SMIs. SDT delineates intrinsic and extrinsic motivations that can simultaneously affect behavior [19]. There is an imperative need for research that transcends existing boundaries to offer a more comprehensive assessment of the profound influence SMIs have on their followers.

In this study, the ultimate outcome of exposure to SMIs is conspicuous consumption, as followers aim to acquire to impress others and signal their financial independence [20]. Based on the lack of a guiding framework for SMIs in the literature, this study endeavors to explore how SMIs can influence their followers' conspicuous consumption. What journey do followers undergo after exposure to SMIs on social media? What factors can mediate the exposure of SMIs and the conspicuous consumption of followers? To address these questions, this study underscores a novel model featuring a dual path to enhance the comprehension of the influence of SMIs’ exposure on conspicuous consumption. The elucidation of SMIs' impact revolves around the aspiration to emulate materialistic tendencies in acquiring conspicuously endorsed products.

While previous research only focuses on a direct effect of SMIs on their followers’ buying decisions, a broader view is needed here to gain more insight on this phenomenon. By combining the SOR framework and SDT theory, this study will elucidate followers' motivations for engaging in conspicuous consumption after exposure to SMIs, using a dual-path framework called the Intrinsic-Extrinsic Consumption Motivation Model (IECM). The first path, rooted in intrinsic motivation, is where followers' desire to push themselves to engage in conspicuous consumption under the intermediation of desire to mimic and materialism. To thoroughly explore this avenue, it is imperative to closely examine the direct and indirect effects of exposure to social media influencers, the desire to mimic, materialism, and conspicuous consumption (Hypotheses 1–4). The other path focuses on the extrinsic, where followers actively engage in upward comparisons with SMIs, contend with the FOMO, and subsequently participate in conspicuous consumption (Hypotheses 5–8). The examination of the relationship between Social Media Influencers (SMIs), social comparison, Fear of Missing Out (FOMO), and conspicuous consumption, as explored in each hypothesis, offers invaluable insights into consumers' decision journeys.

This study unequivocally posits the desire to mimic, social comparison, materialism, and FOMO as pivotal mediators in the intricate relationship between exposure to SMIs and conspicuous consumption. Despite the acknowledged importance of FOMO in deciphering customer behavior, a substantial gap in our understanding persists regarding its relationship with conspicuous consumption and its potential role in SMIs. The anxiety of missing out on trendy and noticeable products drives customers' desire for conspicuous consumption when they aim to improve their social status. On the other hand, individuals learn about materialistic values through content showcasing renowned personalities on social media [21]. Therefore, this study addresses the existing research gap by integrating FOMO and materialism as pivotal mediators in the formation of customer intentions regarding conspicuous consumption.

By undertaking this integration, the research makes a substantial contribution to the contemporary literature on SMIs, FOMO, materialism, and conspicuous consumption. It enhances our understanding by offering a comprehensive insight into the interrelated dynamics of these factors. This empirical model has several theoretical and practical implications for researchers, marketers, and SMIs in developing effective marketing plans. It contributes to the literature on customer behavior when they connect with SMIs in the conspicuous consumption context, asserting that there is a scarcity of both empirical research and theoretical models in this domain. The findings not only extend the applicability of the Stimulus-Organism-Response (SOR) framework and the Self-Determination Theory (SDT) to this emerging field but also elucidate the involvement of the four mediating variables. Moreover, this study offers valuable insights for SMIs, businesses, and social media users. Based on these results, brands and SMIs stand to enhance their understanding of social media users' reactions, enabling them to tailor their appearances and content for a more effective impact on their target audience.

The remainder of this paper is organized as follows. The next section brings more detail of the desire to mimic, materialism, social comparison, FOMO, and conspicuous consumption and provides an overview of the previous research on the relationship between these constructs. Next, the methodology section describes the procedures used to collect the data and measure the constructs. The subsequent section reports the results of this study, including the measurement model used to examine the validity, reliability, model index, and testing of the hypotheses. The final section discusses the results based on the findings and conclusions related to theoretical and practical implications and recommends future research based on the limitations of this study.

## 2 Literature review

### 2.1 Social media influencers

SMIs are content creators who typically set new trends and share their experiences about products, lifestyles, and campaigns on social media platforms [18]. SMIs are micro-celebrities, "social media stars" [1], who share their daily lives on social media to interact with their followers. SMIs create attractive online images by communicating authentic stories with the support of videos, photos, activities, and benefits from brands [22]. Therefore, SMIs are perceived to be more specific, engaged, and friendly to audiences than traditional celebrities [23,24].

Moreover, SMIs possess unique advantages in accessing and endorsing the latest products or services within their areas of interest, often positioning themselves as trendsetters and experts [25]. Therefore, SMIs gain the advantage of having insight into such products, fitting them into their lives before sharing them with their viewers [23]. Their frequent display of trendy possessions reinforces this image, further enhancing their appeal to followers seeking to emulate their lifestyles [24]. The careful disposition of interesting online characters helps SMIs achieve a huge number of followers who have to be continuously engaged with SMIs on social media [26]. Previous research has explored the influence of SMIs on followers' purchase decisions based on their endorsements, such as perceived credibility, expertise, and attractiveness directly affect audiences' buying intention [[12], [11], [13], [14]]. However, what is the journey that followers go through when exposed to SMIs, leading them to conspicuous consumption? What factors affect followers’ decisions when engaging with SMIs' content that can motivate them to buy the conspicuous trendy products that SMIs endorse? This gap needs to be addressed in order to understand the importance of exploring the connection between SMIs and their followers to gain deeper insights into the processes driving conspicuous consumption behaviors.

#### 2.1.1 Conspicuous consumption

Conspicuous consumption is contingent on symbolic, scarce, and cultural capital goods that portray a self-image [27]. Veblen [20] defines conspicuous consumption as spending time and money on unnecessary and ineffective products to express self-image in society. Expensive and luxury products can serve as a barrier to product acquisition, thereby increasing their attractiveness and conferring status, wealth, honor, and esteem upon the individual [28]. Gierl and Huettl [29] identify three types of conspicuous consumption: ostentation and symbolism (utilizing and purchasing material items that can signal a high social status), uniqueness (distinguishing consumers to showcase their uniqueness relative to others), and social conformity (utilizing products to enhance the likelihood of belonging to exclusive social groups.

Previous research has found that conspicuous consumption results from individual emotions and social needs. The emulation of the social group in a higher position in the hierarchy [30], a need for uniqueness [31], or countervailing need for conformity [32] affect customers' purchases of conspicuous goods. Conspicuous consumption is also found to be influenced by social media usage [33]. Moreover, it is a prevalent concept in studies investigating customers’ purchasing decisions centered on the personal self in consumption scenarios. Previous studies have effectively established the connection between personal identity and the purchase of conspicuous goods, indicating that individuals express themselves through the acquisition of branded items that have images compatible with their own [34]. Furthermore, customers' decisions to purchase conspicuous goods are influenced by prominent figures, such as key opinion leaders [35], celebrities in mass media [36], or social media influencers [37].

#### 2.1.2 Theoretical foundation

This study proposes a framework based on the SOR framework and SDT theory to address unexplored research questions. Previous studies have utilized the SOR framework to comprehend the behavior resulting from environmental factors, referred to as stimuli [16,17]. These stimuli impact the organism and subsequently prompt behavioral responses. This three-part framework allows for model formulation, incorporating layers of emotional and cognitive mediation rather than a direct causal relationship between stimulus and response behaviors. In this study, SMIs serve as stimuli that activate consumers, necessitating a response before engaging in behaviors. Therefore, when social media users are exposed to SMIs, their progression to purchasing decisions is not immediate; instead, intermediary steps are implicated, including the desire to mimic, materialism, social comparison, and FOMO. These organism factors motivate followers toward conspicuous consumption, the response.

In addition, the SDT, a renowned theory concerning consumers' motivations, is integrated with the SOR framework to enhance understanding of this process. SDT suggests that people's well-being depends on satisfying their basic psychological needs [19]. Based on this theory, intrinsic and extrinsic motivation are two types of motivation that affect behavior [19]. This study propses the IECM model, comprising two pathways. The first path is intrinsic motivation, which refers to actions based on needs and beliefs rather than external rewards or pressures. Exposure to SMIs triggers followers to amplify their desire to mimic, foster materialistic thinking, and ultimately engage in conspicuous consumption. Conversely, the second extrinsic path delineates the impact of extrinsic motivation on followers' reactions to SMIs' content on social media. Defined as engaging in an activity with the expectation of obtaining something in return or avoiding something unpleasant, extrinsic motivation in this context prompts followers to engage in social comparison, leading to the phenomenon of FOMO and subsequent conspicuous consumption.

### 2.2 The mediation role of the desire to mimic and materialism

The desire to mimic SMIs refers to the wish among followers to refine personal traits to better resemble their preferred SMIs [38]. Followers endeavour to mimic SMIs' features to maintain or enhance their identity after deep and long-term exposure to that SMI. Followers consider SMIs as their idealized self-images and try to impersonate SMIs by using SMI-endorsed products. According to Kelman [39], opinion change occurs when individuals believe they can achieve favourable results from the endorser's source. Followers emulate SMIs by accepting the influence and establishing or maintaining a self-identifying relationship with the influencers that becomes part of the follower's self-image.

Materialism refers to the degree of belief that acquisition and possession indicate a happy and successful life [40]. This concept refers to the belief that happiness stems from material fulfillment, whereas conspicuous consumption refers to purchasing products in the presence of wealth. Materialism is much more popular on social media, where people are easily exposed to possession-oriented posts by others [41]. Long-term exposure to SMIs incentivizes individuals to seek materialistic values [42]. A person imitating famous people has been shown to attach much significance to the role of possessions in life [43]. Socialization in online contexts inspires individuals to follow social standards concerning materialistic possessions to gain admission into a social group. Therefore, social media is the most suitable environment for materialism in which people show off their possessions to prove their social worth [41].

### 2.3 The mediation role of social comparison and FOMO

Social comparison refers to the sensitivity experienced when comparing others’ behavior, the degree of uncertainty about oneself and interest in reducing self-uncertainty [44]. Social media users gain self-knowledge by comparing themselves with others, including their thoughts, feelings, and performance. Individuals are even more noticeable and conspicuous in online contexts than in offline contexts [45]. Individuals compare themselves to people enjoying a higher socioeconomic status [46]. Exposure to superiors on social media can enhance upward social comparison [47]. Social media users prefer to compare themselves to those they consider better off. SMIs generally appear as trendy and highly fashionable people with extravagant lifestyles [48]. Thus, SMIs are the primary object for their followers to compare when they connect to them on social media.

FOMO refers to the pervasive apprehension that others may have rewarding experiences when absent [49]. FOMO is usually accompanied by anxiety about social exclusion and the desire to connect with others' activities. Thus, FOMO is the feeling of social media users that their peers do or own more things. Feelings of ostracism and social exclusion are powerful driving forces of FOMO among those with anxiety and low self-esteem [50]. Anxiety is formed through the realization that others enjoy more benefits and rewards. Self-esteem is considered a dimension of FOMO that predicts the conspicuous consumption of branded fashion accessories [51]. FOMO is often linked to the expression “keeping up with the Joneses,” which implies that individuals try to get what others have and do what they do, such as buying expensive cars and clothes that they cannot afford, to show that they are as good as others [52].

### 2.4 Research framework

**Fig. 1:**
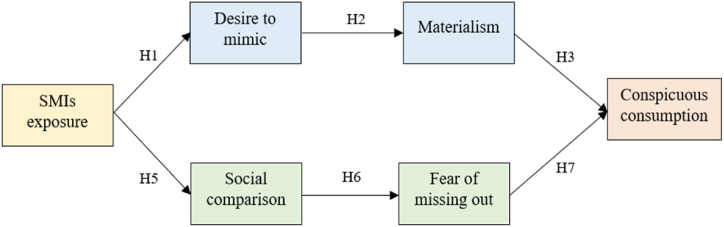
Theoretical framework.

Fig. 1 illustrates the framework of this study with a dual path from SMIs exposure to conspicuous consumption. In the first pathway, intrinsic motivation, both the desire to mimic and materialism, are interconnected. These elements exist among followers and are triggered by exposure to SMIs. SMIs serve as significant influencers for followers seeking trendy products and services. Admiration for SMIs is widespread because they showcase the trendiness and quality of life that followers aspire to Ref. [25]. Through exposure to SMIs, followers seek to emulate them and experiment with endorsed suggestions to enhance their self-image [53]. Ki and Kim [38] claimed that once followers are satisfied and trust SMIs’ endorsements, they want to mimic SMIs and purchase the endorsed products used by those SMIs.

Additionally, SMIs portray idealized selves, and their identities are inextricably linked to the possessions surrounding them and their experiences, leading others to aspire to their lifestyle [54]. Exposure to SMIs makes followers more likely to anticipate success, happiness, and status. Research by Rasmussen, Riggs and Sauermilch [55] on SMIs indicated that those with high levels of materialism have the potential to desire fame, adjust their behaviors, and purchase conspicuous products to be noticed by others. Therefore, this study proposes the following hypothesis:

Hypothesis 1 Exposure to SMIs is positively related to the desire to mimic SMIs.

Hypothesis 2 Desire to mimic SMIs is positively related to materialism.

Hypothesis 3 Materialism is positively related to conspicuous consumption.

Hypothesis 4 Desire to mimic and materialism mediate the relationship between exposure to SMIs and conspicuous consumption.

In the second pathway, social comparison and FOMO are extrinsic motivations formed under social pressure and the fear of being left behind in the current social life. Followers are influenced by the desire within themselves and the urge for social status, social trends, or potential rewards and experiences. According to Festinger [56], individuals form a self-image based on how they compare themselves with others. Followers of SMIs' endorsed products are more likely to engage in social comparisons in social media contexts [57]. Individuals who are uncertain are more likely to compare themselves with those who are better off [58].

Moreover, FOMO results from regular exposure to others' information and comparisons [59]. Followers have a strong desire to keep up with the latest trends and must be well-acquainted with what happens on social networks [49]. SMIs are the primary sources on which their followers depend to obtain social approval because they symbolize trendiness and success [48]. Owing to social comparison, the FOMO of endorsed products motivates people to consume them when their self-esteem is threatened. As a result, they try to buy products or services to avoid social exclusion [60]. Intense FOMO of products and experiences can encourage people to consume products to attain and retain their social status. Basic needs such as self-esteem, the need to keep up with trends, and the fear of falling behind are dimensions of FOMO [61]. The lack of these needs is only fulfilled by gaining products consumed by the referent group to obtain a social impression [62]. Conspicuous consumption enhances needs, improves self-esteem [55], and attracts attention through self-concentration to impress others [63]. Based on these assertions, the following hypotheses are formulated:

Hypothesis 5 Exposure to SMIs is positively related to social comparisons to SMIs.

Hypothesis 6 Social comparison to SMIs is positively related to FOMO on endorsed products.

Hypothesis 7 FOMO on endorsed products is positively related to the desire for conspicuous consumption.

Hypothesis 8 Social comparison and FOMO mediate the relationship between SMIs exposure and conspicuous consumption.

## 3 Research methodology

### 3.1 Sample

The study utilized a sample size of 300 US social media users recruited via an anonymous survey through the Amazon Mechanical Turk (MTurk) platform in January 2021. The sample size was estimated using the method outlined by Field, Miles and Field [64] for a survey incorporating mediation and moderation effects. The survey instrument was carefully designed to protect the anonymity and confidentiality of participants, and no personally identifiable information was collected. Participants were informed of the voluntary nature of their participation and consent was obtained from all respondents, and the study complies with all ethical regulations. Respondents following beauty influencers were included in this research to explore their perceptions concerning SMIs and their agreement with statements about exposure to SMIs, desire to mimic, materialism, social comparison, FOMO, and conspicuous consumption. Beauty influencers are the most similar and popular type of SMIs [65], so they were selected as the research focus. The participants who reported not being exposed to influencers before were excluded from this study. In the end, the sample size was 272, including 133 female respondents (48.9 %) and 139 male respondents (51.1 %). The respondents ranged from 18 to 50 years, with 44.9 % in their 30s.

### 3.2 Measurement

This study utilized measurements from previous research and revised them to match the context. Altogether, twenty-nine questions were used, including exclusive questions about favourite beauty influencers, social platforms used to connect with favourite influencers, demographic questions concerning age and gender, and questions targeting six specific constructs. Finally, a seven-point Likert scale was used to measure the level of agreement among statements (ranging from 1 “*strongly disagree”* to 7 “*strongly agree”*).

Exposure to SMIs was derived from Ross, Orr, Sisic, Arseneault, Simmering and Orr [66] questionnaire wherein the context was changed to that of a social media influencer to fit the study's purpose. The scale of desire to mimic was derived from Kasser, Ryan, Couchman and Sheldon [48] study. Upward social comparison to SMIs scale was devised based on Solberg, Diener, Wirtz, Lucas and Oishi [67] scale. We employed eight statements from the FOMO scale of Good and Hyman [60] to measure FOMO. This research utilized a three-item scale developed by Richins [68] to test beliefs about material possession. Conspicuous consumption scale was conceived based on the study of O'Cass and McEwen [69].

#### 3.2.1 Data analysis

Drawing on the SOR framework, SDT theory, and the relationships among constructs established in previous studies, this research utilizes the Amos 23.0 program to analyze data via measurement and structural models. This choice is made because CB-SEM (covariance-based structural equation modeling) is particularly suitable for confirmatory analysis, relying on carefully developed measures grounded in theoretical foundations [70]. Moreover, this method is widely employed in data analysis due to its ability to integrate regression and confirmatory factor analysis. The measurement model was designed to assess the model's fit, reliability, validity, and multicollinearity among the constructs, ensuring the reliability and validity of the data. The structural equation model was utilized to test and estimate relationships among variables, thereby addressing both the direct and indirect hypotheses of this study.

## 4 Results

### 4.1 Measurement model

The measurement model was tested using confirmatory factor analysis (CFA). The chi-square test had acceptable values *(ꭓ*^*2*^ *[256]* = 379.831, *p* < 0.05, *CMIN/DF* = 1.484). Model fit was assessed using the goodness of fit (GFI), Tucker-Lewis Index (TLI), comparative fit index (CFI), and root mean square error approximation (RMSEA) [71]. The *GFI* = 9.02, *TLI* = 0.951, *CFI* = 0.958, and *RMSEA* = 0.042 demonstrated that the model represented a good model fit. According to Pesämaa, Zwikael, Hair and Huemann [72], the factor loading should be higher than 0.50 but not greater than 0.95. The composite reliability (CR) should exceed 0.6, and the average variance extracted (AVE) should exceed 0.5. The square root of the AVE should have the highest value compared to the correlations between variables. Table 1 illustrates that the lowest factor loading value was 0.617, the lowest CR value was 0.766, the lowest AVE was 0.501, and the lowest Cronbach's α was 0.74. As displayed in Table 2, the discriminant validity of this study is satisfactory, and all estimated path coefficients are statistically significant. Therefore, all the requirements were met, confirming the validity and reliability of this study.

**Table 1:** Scale reliabilities.

Item	M	SD	Factor loading
Exposure to social media influencers (CR = 0.797, AVE = 0.567, Cronbach's α = 0.792)			
Connecting with influencers through SNS platforms is part of my everyday activity.	5.504	1.190	0.712
I dedicate a part of my daily schedule to connect with influencers through SNS platforms.	5.136	1.355	0.810
I feel I am part of the influencers' community on SNS platforms.	5.305	1.302	0.734
Desire to mimic social media influencers (CR = 0.766, AVE = 0.526, Cronbach's α = 0.792)			
I want to be as stylish as influencers.	5.768	0.980	0.827
I want to be as trendy as influencers.	5.860	0.954	0.617
I aspire to the lifestyle of influencers.	5.739	0.980	0.716
Social comparison (CR = 0.834, AVE = 0.501, Cronbach's α = 0.831)			
Influencers can afford a better dwelling (apartment, house) than me.	5.820	1.031	0.671
Influencers can afford better food and drink than me.	5.787	1.009	0.646
Influencers can afford more expensive entertainment than me.	5.798	1.002	0.766
Influencers can afford better and more clothes than me.	5.835	1.062	0.696
Influencers can afford to pay school expenses more easily than me.	5.878	0.975	0.754
Fear of missing out (CR = 0.898, AVE = 0.525, Cronbach's α = 0.899)			
I'm afraid I will feel sorry I didn't buy endorsed products later.	5.662	1.204	0.725
I will worry that I'm missing out on endorsed products.	5.805	0.984	0.732
I will worry that other people are having more rewarding experiences than me by using endorsed products.	5.743	1.076	0.739
I feel concerned that other people are having more fun with endorsed products.	5.688	1.006	0.715
I will feel left out of trends if I don't have endorsed products.	5.735	0.935	0.737
I will feel sorry that I didn't experience endorsed products.	5.702	1.043	0.716
I will feel anxious about not having endorsed products.	5.643	1.121	0.673
I will feel bothered that I missed an opportunity to use endorsed products.	5.640	1.060	0.756
Materialism (CR = 0.774, AVE = 0.536, Cronbach's α = 0.764)			
I admire people who own expensive homes, cars, and clothes.	5.504	1.236	0.811
The things I own say a lot about how well I'm doing in life.	5.577	1.107	0.753
I'd be happier if I could afford to buy more things.	5.441	1.246	0.619
Conspicuous consumption (CR = 0.769, AVE = 0.526, Cronbach's α = 0.768)			
I want to buy products to be noticed by others.	5.706	0.958	0.710
I want to buy products because of the presence of others.	5.647	0.905	0.699
I want to buy products to gain respect.	5.673	0.960	0.765

Notes: SD, standard deviation; λ, Factor loading; CR, composite reliability; AVE, average variance extracted.

**Table 2:** Correlation matrix and discriminant assessment.

	EX	DM	SC	FOMO	MA	CON
Exposure to SMIs (EX)	**0.753**					
Desire to mimic SMIs (DM)	0.696***	**0.725**				
Social comparison (SC)	0.237**	0.407***	**0.708**			
FOMO	0.301***	0.308***	0.482***	**0.724**		
Materialism (MA)	0.563***	0.721***	0.509***	0.395***	**0.732**	
Conspicuous consumption (CON)	0.554***	0.509***	0.270***	0.392***	0.606***	**0.725**

Note: ***p < 0.001; **p < 0.01; squares of AVE are shown in bold on the diagonal.

To check for multicollinearity among the variables, we ran a collinearity test. According to Ringle, Wende and Becker [73], multicollinearity is ruled out if the tolerance values exceed 0.2 and the variance inflation factor (VIF) is less than 5. Table 3 shows that the lowest tolerance value in this study was 0.564, and the highest VIF value was 1.774. Therefore, multicollinearity was not a problem in this study.

**Table 3:** Tolerance and VIF.

Variables	Tolerance	VIF
Exposure to SMIs	0.688	1.453
Desire to mimic	0.571	1.752
Social comparison	0.588	1.702
Materialism	0.564	1.774
FOMO	0.810	1.234

### 4.2 Structural model

The model fit of the structural model was first tested to confirm its suitability of the model. The chi-square test was significant, with *ꭓ*^*2*^ *(269)* = 495.728, *p* < 0.05, and *CMIN/DF* = 1.843. Other model fit indicators were acceptable, with *GFI* = 0.873, *CFI* = 0.923, *TLI* = 0.914, and *RMSEA* = 0.056. These findings indicate that our hypothesized model is valid.

As hypothesized, exposure to SMIs positively affected the desire to mimic and social comparison, supporting H1 and H5 *(ꞵ* = 0.725 and *ꞵ* = 0.329, respectively). We can conclude that the more individuals are exposed to SMIs through SNS platforms, the more SMIs they imitate, with a higher tendency to make social comparisons. The first path of customers’ underlying processes revealed that materialism mediated the relationship between the desire to mimic and conspicuous consumption. Hence, H2 and H3 are accepted. This implies that the desire to mimic SMIs is more likely to evoke materialism and urge consumers to consume conspicuous products. Another path in this process is the mediating role of FOMO. H6 was supported (*ꞵ* = 0.493), indicating that FOMO results from individuals comparing themselves to SMIs. Once individuals fear missing out on endorsed products, they are more likely to buy them, supporting H7 *(ꞵ* = 0.208). These findings are summarized in Table 4.

**Table 4:** Hypotheses of the proposed theoretical model.

Hypothesis	Estimate	*t*-Test	P	Supported
H1. Exposure to SMIs → Desire to mimic SMIs	0.725	7.869	***	Yes
H2. Desire to mimic SMIs → Materialism	0.747	7.191	***	Yes
H3. Materialism → Conspicuous consumption	0.567	6.185	***	Yes
H5. Exposure to SMIs → Social comparison	0.329	4.392	***	Yes
H6. Social comparison → FOMO	0.493	6.633	***	Yes
H7. FOMO → Conspicuous consumption	0.208	3.123	**	Yes

Note: ***p < 0.001; **p < 0.01.

The 95 % bias-corrected confidence intervals with 5000 bootstrapped samples were used to test the mediation effects of desire to mimic, materialism, social comparison, and FOMO on the relationship between exposure to SMIs and the desire for conspicuous consumption. The relationships with the upper and lower bounds, which should not include zero, were accepted [74]. The results of the indirect effects are displayed in Table 5. All indirect paths in this study were significant, and social comparison was the mediator of exposure to SMIs and FOMO (CI: [0.041, 0.273]). FOMO was confirmed to mediate the relationship between social comparison and conspicuous consumption (CI: [0.032, 0.167]). Both mediated the effects of exposure to SMIs on conspicuous consumption (CI: [0.005, 0.056]). Moreover, the desire to mimic SMIs and materialism mediated the relationship between exposure to SMIs and conspicuous consumption (CI: [0.125, 0.317]). Therefore, H4 and H8 are supported.

**Table 5:** Mediation effects.

Parameter	Estimate	Lower	Upper	P	Conclusion
EX → SC → FOMO	0.148	0.041	0.273	***	Indirect effect
EX → SC → FOMO → CON	0.022	0.005	0.056	***	Indirect effect
SC → FOMO → CON	0.086	0.032	0.167	***	Indirect effect
EX → DM → MA	0.422	0.308	0.588	***	Indirect effect
EX → DM → MA → CON	0.197	0.125	0.317	***	Indirect effect
DM → MA → CON	0.423	0.260	0.660	***	Indirect effect

Note: ***p < 0.001; EX = exposure to influencers; DM = desire to mimic social media influencers; SC = social comparison; MA = materialism; CON, Conspicuous consumption.

**Fig. 2:**
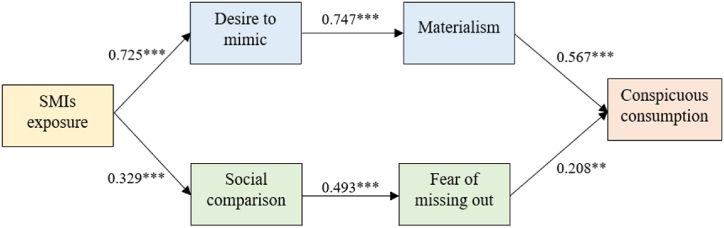
Research findings.

Fig. 2 presents the overall results of this study.

## 5 Discussion

This study proposes and tests the IECM model, which elucidates the influence of SMIs on followers' conspicuous consumption behavior, with the mediating roles of desire to mimic, materialism, social comparison, and FOMO. The empirical test yielded significant insights into understanding the relationship between exposure to SMIs and conspicuous consumption. The IECM model is a novel research framework formed based on the SOR framework and SDT theory, which integrates both intrinsic motivations (such as the desire to mimic and materialism) and extrinsic motivations (including social comparison and FOMO). The result indicates that SMIs strongly affect customers' desire for conspicuous consumption. When exposed to SMIs, customers are more likely to consume conspicuous products because of SMIs' aspirations and belief in enhancing their self-image. The effect of SMIs on conspicuous consumption is explained using the SOR framework with intrinsic and extrinsic motivations from SDT. Exposure to SMIs encourages followers to engage in conspicuous consumption through intrinsic motivation as a desire to mimic materialism. These results confirm that exposure to SMIs can make followers want to mimic them, leading to higher materialism and conspicuous consumption. Individuals who connect with and dedicate their daily schedules to following their favourite SMIs are likelier to mimic them. The results show that individuals who want to be stylish and trendy and admit SMIs’ lifestyles consider material acquisition one of the most vital measurements of social success. Frequent exposure to SMIs builds bonds between followers and SMIs, leading followers to believe they need to learn from SMIs to improve their self-image [26]. Imitating SMIs increases customer materialism, making customers want to possess more endorsed, conspicuous products.

Materialism is crucial in the relationship between exposure to SMIs and conspicuous consumption. SMIs appear on social media with engaging life stories and conspicuous products that attract their audience and make them want to live like them [29]. This study verifies that consumers who admit that others with high-quality possessions purchase more products. A deep relationship with SMIs encourages people to gain respect by owning material possessions, which is how they gain social positions [24]. Materialists use products, services, and experiences to express prestige and status. Therefore, the desire to mimic and materialism are intrinsic reactions of followers when they are exposed to SMIs and buy conspicuous products.

In addition, extrinsic motivation for social comparison and FOMO mediated the relationship between exposure to SMIs and conspicuous consumption. This study reveals that individuals exposed to SMIs prefer to make upward social comparisons with SMIs. Social media users are more likely to compare themselves with individuals who are better at self-evaluating [46]. These comparisons focus on lifestyle and material aspects. SMIs represent a high standard of life and good taste [38]. To enhance their social status, people compare themselves with SMIs to realize their current position [57]. This comparison makes consumers feel anxious, making them want to be better, more entertained, and enjoy better outcomes than others, an expression of FOMO. Thus, this study found that social comparison is a medium of exposure to SMIs to feel FOMO.

The important finding of this study is that FOMO mediates the relationship between SMIs exposure and conspicuous consumption. Little effort has focused on FOMO's role in social media contexts and its power in conspicuous consumption. In this study, FOMO is the feeling that emerges after individuals self-evaluate, fearing that they will miss out on trends. They try to consume products and services to attain and maintain their social status and avoid distress. According to previous research, when individuals compare themselves to others, their self-esteem is threatened, and they recover their self-image by consuming products to obtain a social impression [62]. Conspicuous consumption is the go-to choice for enhancing needs, improving self-esteem, and attracting attention [63]. The greater the fear of individuals, the higher their conspicuous consumption of the endorsed products. This study empirically tested the motivation roles of social comparison and FOMO as the organism of followers when they are exposed to SMIs.

### 5.1 Theoretical implications

This study contributes to the literature on the SOR framework, SDT, SMIs, FOMO, materialism, and conspicuous consumption. Despite the association between SMIs' endorsements and conspicuous products, the literature has yet to comprehensively explain this phenomenon. Previous research on SMIs still primarily focuses on the influence of SMIs' endorsements on buying intention [57,75]. To address this gap, this empirical test elucidates the underlying process of customer journeys from exposure to SMIs to conspicuous consumption, delineating dual pathways. This study contributes to the literature on customer behavior and SMIs by demonstrating the significant influence of SMIs on customers’ buying decisions, particularly concerning conspicuous products.

The integration of the SOR framework and SDT offers a more comprehensive perspective on the phenomenon of SMIs within the realm of conspicuous consumption. Previous studies have primarily concentrated on the endorsements of SMIs, which directly impact followers' purchasing intentions [[12], [11], [13], [14]]; however, this study proposes a narrative incorporating the mediating effects of the desire to mimic, materialism, social comparison, and FOMO. The SOR framework was applied to explain the followers' journey from SMIs’ exposure and conspicuous consumption through the mechanisms of desire to mimic, social comparison, materialism, and FOMO. Furthermore, to gain deeper insights into this phenomenon, this study employs the SDT theory to propose a dual model. Intrinsic and extrinsic motivation has been applied in education, organization, and consumer decision-making behavior [[76], [77], [78]] but they have not been utilized to elucidate the context of SMIs or FOMO previously. Therefore, this study extends the literature on the SOR framework and SDT to encompass SMIs and the context of conspicuous consumption.

Moreover, FOMO has been utilized to motivate customers to contemplate and purchase products or brands [49], yet previous research has solely examined it to assess customer buying intention [57,60,79]. This study examines FOMO in a novel context as a catalyst in the customer's purchasing journey when exposed to SMIs in conspicuous consumption. The results demonstrate that FOMO significantly influences conspicuous consumption behavior under the influence of SMIs. This study enhances the literature on FOMO and offers another perspective for researchers to explore conspicuous consumption in the context of SMIs.

Lastly, we examine the relationships between social comparison, materialism, and the desire to mimic SMIs in situations of conspicuous consumption. While previous research has used the imitation of celebrities as a precursor to materialism [80,81], few studies have explored the role of the desire to mimic SMIs in the context of conspicuous consumption. Furthermore, this study investigates the impact of social comparison with SMIs on conspicuous consumption under the influence of SMIs. By integrating these concepts, we demonstrate the diverse scenarios that may arise when customers contemplate purchasing conspicuous products. Additionally, we contribute to the literature on social comparison, materialism, the desire to mimic SMIs, and conspicuous consumption by expanding its scope.

#### 5.1.1 Practical implications

This study also offers several noteworthy implications for practitioners, including social media users, SMIs, and marketers. Firstly, it elucidates how SMIs can influence conspicuous consumption. Therefore, social media users need to recognize the crucial factors that influence their purchasing decisions, as highlighted by Fox and Moreland [82], given that many social media users are unaware of how SMIs influence them and manipulate their behaviors.

Additionally, this study suggests that employing SMIs in marketing practices is an effective strategy for promoting businesses, particularly for luxury or established brands. Exposure to SMIs can heighten followers' desire for conspicuous products. Random social comparisons outside the product's domain are less likely to be perceived as promotional messages and more likely to elicit consumer counter-reactions [83]. Hence, marketers can collaborate with SMIs to influence customers into purchasing products and services. Leveraging their intimate understanding of their followers, SMIs can anticipate which products their followers would favor and adeptly craft and disseminate marketing messages to the target audience [84]. They can promote commercial entities, such as products and brands, by establishing a logical connection to their identity and presenting it as a natural extension of their existing organic content [85]. Therefore, customers place greater trust in their recommendations when SMIs share authoritative experiences, leading them to receive brand messages from SMIs without hesitation.

Moreover, conspicuous consumption arises from materialistic behavior and FOMO regarding rewarding or trendy products. Marketers should adeptly incorporate FOMO appeal into marketing campaigns, compelling customers to purchase these products to avoid missing out on their benefits (e.g., limited edition, scarcity of items). Additionally, marketers should emphasize the 'prestige,' 'social inclusion,' and 'trendiness' that customers may derive from purchasing items (e.g., cosmetic products containing gold or expensive ingredients, distinctive packaging). Marketers should ensure that their products are well-suited for consumption purposes and capable of projecting a distinct and unique image to users, given that consumers primarily utilize a brand's products to elevate their social status. Marketers should refine their marketing strategies and cultivate brand identities that resonate with the prevailing self-perceptions of consumers.

#### 5.1.2 Limitation and future research

Some limitations of this study could offer valuable insights for designing future research. Firstly, this study focused on beauty influencers as the research subjects. However, other types of SMIs, such as fashion and gaming influencers, may possess distinct characteristics that could yield more robust results. Future research could address this limitation by broadening the sample beyond specific criteria. Diversifying the sample across different domains could offer a more comprehensive understanding of how SMIs influence conspicuous consumption across various sectors. Moreover, while this study explored the impact of exposure to SMIs on conspicuous consumption, future research could investigate this finding in terms of customers’ purchase intention, impulse buying behavior, or even acceptance of marketing campaigns. The study treated SMIs as a homogeneous group, overlooking potential variations based on audience size. Future research could delve into the nuanced effects of SMIs with different follower counts. SMIs can be categorized into micro or macro influencers based on their number of followers [78], examining whether an SMI with a larger audience can exert a more significant influence on their followers' reactions compared to an SMI with a smaller following. Furthermore, a comparative analysis of SMIs with distinct characteristics, such as follower engagement strategies, posting frequency, or content types, would be beneficial. Understanding how these factors contribute to conspicuous consumption desires or FOMO experiences can provide practical insights for SMIs and marketers seeking to customize their strategies for specific audience segments.

Additionally, the sample size comprised only 272 valid respondents from the MTurk platform in the US. This limited sample size and sampling method constrain the reliability and generalizability of the study findings. Future research should account for cultural differences concerning conspicuous consumption, the significance of material possessions, and social influences, which may produce varied outcomes. Therefore, it is advisable for future studies to apply this model to diverse cultural contexts for more precise market insights. Moreover, employing larger sample sizes would enhance the statistical power of the results. Furthermore, this study may potentially suffer from common method bias, as it relied solely on a self-report questionnaire to measure all constructs. This approach may have induced respondents to adopt a consistent response style, thereby inflating correlations among variables. To mitigate this issue, future research could employ multiple methods to measure the constructs.

## 6 Conclusion

Drawing upon the SOR framework and SDT, this study introduces the IECM model, which elucidates the influence of SMIs on conspicuous consumption through dual pathways. In this model, intrinsic factors such as the desire to mimic and materialism assume a pivotal role, as followers emulate SMIs following exposure to their content, thereby eliciting materialistic desires that ultimately manifest in conspicuous consumption behaviors. Additionally, the model highlights the extrinsic pathway, in which exposure to SMIs leads to social comparison and Fear of Missing Out (FOMO), resulting in conspicuous consumption. By pioneering the integration of the SOR framework and SDT within the context of SMIs, this study introduces a novel framework that can better elucidate followers’ conspicuous consumption behavior and the applicability of these theoretical frameworks. Moreover, the findings offer valuable insights for marketers and SMIs, providing guidance on leveraging these insights to effectively engage and attract consumers in the digital landscape.

## Funding

This work was supported by the 2024 10.13039/501100002649Yeungnam University Research Grant.

## Ethic statement

All participants provided informed consent to participate in the study. The survey explicitly committed to preserving respondents' privacy by utilizing their data solely for scientific research purposes, refraining from any other use, and ensuring the anonymity of participants.

## Data availability statement

https://doi.org/10.6084/m9.figshare.21786296.v1.

## CRediT authorship contribution statement

**Thi Cam Tu Dinh:** Writing – original draft, Methodology, Formal analysis, Conceptualization. **Yoonjae Lee:** Writing – review & editing, Investigation, Conceptualization.

## Declaration of competing interest

The authors declare that they have no known competing financial interests or personal relationships that could have appeared to influence the work reported in this paper.

## References

[1] Hudders L., De Jans S., De Veirman M. (2021). The commercialization of social media stars: a literature review and conceptual framework on the strategic use of social media influencers. Int. J. Advert..

[2] Leban M., Thomsen T.U., von Wallpach S., Voyer B.G. (2021). Constructing personas: how high-net-worth social media influencers reconcile ethicality and living a luxury lifestyle. J. Bus. Ethics.

[3] Tafesse W., Wood B.P. (2021). Followers' engagement with instagram influencers: the role of influencers' content and engagement strategy. J. Retailing Consum. Serv..

[4] Steils N., Martin A., Toti J.-F. (2022). Managing the transparency paradox of social-media influencer disclosures. J. Advert. Res..

[5] Tanwar A.S., Chaudhry H., Srivastava M.K. (2022). Trends in influencer marketing: a review and bibliometric analysis. J. Interact. Advert..

[6] Geyser W. (2024). The state of influencer marketing 2024: benchmark report. https://influencermarketinghub.com/influencer-marketing-benchmark-report/.

[7] Vrontis D., Makrides A., Christofi M., Thrassou A. (2021). Social media influencer marketing: a systematic review, integrative framework and future research agenda. Int. J. Consum. Stud..

[8] Lou C., Kim H.K. (2019). Fancying the new rich and famous? Explicating the roles of influencer content, credibility, and parental mediation in adolescents' parasocial relationship, materialism, and purchase intentions. Front. Psychol..

[9] Sekhon T., Bickart B., Trudel R., Fournier S. (2016). Consumer Psychology in a Social Media World.

[10] Liu S., Jiang C., Lin Z., Ding Y., Duan R., Xu Z. (2015). Identifying effective influencers based on trust for electronic word-of-mouth marketing: a domain-aware approach. Inf. Sci..

[11] Ford J.B. (2022). Advertising message impact on consumers: how to enhance positive affect. J. Advert. Res..

[12] Breves P.L., Liebers N., Abt M., Kunze A. (2019). The perceived fit between instagram influencers and the endorsed brand. J. Advert. Res..

[13] Delbaere M., Michael B., Phillips B.J. (2021). Social media influencers: a route to brand engagement for their followers. J. Advert. Res..

[14] Schouten A.P., Janssen L., Verspaget M. (2020). Celebrity vs. Influencer endorsements in advertising: the role of identification, credibility, and Product-Endorser fit. Int. J. Advert..

[15] Boerman S.C., Müller C.M. (2022). Understanding which cues people use to identify influencer marketing on Instagram: an eye tracking study and experiment. Int. J. Advert..

[16] Laato S., Islam A.K.M.N., Farooq A., Dhir A. (2020). Unusual purchasing behavior during the early stages of the COVID-19 pandemic: the stimulus-organism-response approach. J. Retailing Consum. Serv..

[17] Chan T.K.H., Cheung C.M.K., Lee Z.W.Y. (2017). The state of online impulse-buying research: a literature analysis. Inf. Manag..

[18] Lou C., Yuan S. (2019). Influencer marketing: how message value and credibility affect consumer trust of branded content on social media. J. Interact. Advert..

[19] Ryan R.M., Deci E.L. (2000). Intrinsic and extrinsic motivations: classic definitions and new directions. Contemp. Educ. Psychol..

[20] T. Veblen, The Theory of the Leisure Class, Oxford University Press2009.

[21] Seo E.-J., Park J.-W. (2018). A study on the effects of social media marketing activities on brand equity and customer response in the airline industry. J. Air Transport. Manag..

[22] Childers C.C., Lemon L.L., Hoy M.G. (2019). #Sponsored #ad: agency perspective on influencer marketing campaigns. J. Curr. Issues Res. Advert..

[23] Djafarova E., Rushworth C. (2017). Exploring the credibility of online celebrities' Instagram profiles in influencing the purchase decisions of young female users. Comput. Hum. Behav..

[24] Jin S.V., Muqaddam A., Ryu E. (2019). Instafamous and social media influencer marketing. Market. Intell. Plann..

[25] Aral S. (2011). Commentary—identifying social influence: a comment on opinion leadership and social contagion in new product diffusion. Market. Sci..

[26] Ki C.-W., Cuevas L., Chong S., Lim H. (2020). Influencer marketing: social media influencers as human brands attaching to followers and yielding positive marketing results by fulfilling needs. J. Retailing Consum. Serv..

[27] Roy Chaudhuri H., Mazumdar S., Ghoshal A. (2011). Conspicuous consumption orientation: conceptualisation, scale development and validation. J. Consum. Behav..

[28] Han Y.J., Nunes J.C., Drèze X. (2010). Signaling status with luxury goods: the role of brand prominence. J. Market..

[29] Gierl H., Huettl V. (2010). Are scarce products always more attractive? The interaction of different types of scarcity signals with products' suitability for conspicuous consumption. Int. J. Res. Market..

[30] Trigg A.B. (2001). Veblen, bourdieu and conspicuous consumption. J. Econ. Issues.

[31] Lee M., Bae J., Koo D.-M. (2021). The effect of materialism on conspicuous vs inconspicuous luxury consumption: focused on need for uniqueness, self-monitoring and self-construal. Asia Pac. J. Mark. Logist..

[32] Brewer M.B. (1991). The social self: on being the same and different at the same time. Pers. Soc. Psychol. Bull..

[33] Oh G.G. (2021). Social class, social self-esteem, and conspicuous consumption. Heliyon.

[34] Aaker J.L. (1999). The malleable self: the role of self-expression in persuasion. J. Market. Res..

[35] Zhang L., Zhao J., Xu K. (2016). Who creates trends in online social media: the crowd or opinion leaders?. J. Computer-Mediated Commun..

[36] Law G., Bloyce D., Waddington I. (2020). Sporting celebrity and conspicuous consumption: a case study of professional footballers in England. Int. Rev. Sociol. Sport.

[37] Dinh T.C.T., Wang M., Lee Y. (2023). How does the fear of missing out moderate the effect of social media influencers on their followers' purchase intention?. Sage Open.

[38] Ki C.-W.C., Kim Y.-K. (2019). The mechanism by which social media influencers persuade consumers: the role of consumers' desire to mimic. Psychol. Market..

[39] Kelman H.C. (2006). Interests, relationships, identities: three central issues for individuals and groups in negotiating their social environment. Annu. Rev. Psychol..

[40] Richins M., Dawson S. (1992). A consumer values orientation for materialism and its measurement. J. Consum. Res..

[41] Islam T., Sheikh Z., Hameed Z., Khan Ikram U., Azam Rauf I. (2018). Social comparison, materialism, and compulsive buying based on stimulus-response-model: a comparative study among adolescents and young adults. Young Consum..

[42] Buijzen M., Valkenburg P.M. (2003). The effects of television advertising on materialism, parent–child conflict, and unhappiness: a review of research. J. Appl. Dev. Psychol..

[43] Chan K., Prendergast G. (2007). Materialism and social comparison among adolescents. SBP (Soc. Behav. Pers.): an international journal.

[44] Gibbons F.X., Buunk B.P. (1999). Individual differences in social comparison: development of a scale of social comparison orientation. J. Pers. Soc. Psychol..

[45] Appel H., Gerlach A.L., Crusius J. (2016). The interplay between Facebook use, social comparison, envy, and depression. Current Opinion in Psychology.

[46] Saunders S. (2001). Fromm's marketing character and rokeach values. SBP (Soc. Behav. Pers.): an international journal.

[47] Lup K., Trub L., Rosenthal L. (2015). Instagram #instasad?: exploring associations among instagram use, depressive symptoms, negative social comparison, and strangers followed. Cyberpsychol., Behav. Soc. Netw..

[48] Kasser T., Ryan R.M., Couchman C.E., Sheldon K.M. (2004). Materialistic Values: Their Causes and Consequences, Psychology and Consumer Culture: the Struggle for a Good Life in a Materialistic World.

[49] Przybylski A., Murayama K., DeHaan C., Gladwell V. (2013). Motivational, emotional, and behavioral correlates of fear of missing out. Comput. Hum. Behav..

[50] Abel J., Buff C., Burr S. (2016). Social media and the fear of missing out: scale development and assessment. J. Bus. Econ. Res..

[51] Souiden N., M'Saad B., Pons F. (2011). A cross-cultural analysis of consumers' conspicuous consumption of branded fashion accessories. J. Int. Consum. Market..

[52] Christen M., Morgan R.M. (2005). Keeping up with the Joneses: analyzing the effect of income inequality on consumer borrowing. Quant. Market. Econ..

[53] Boon S., Lomore C. (2001). Admirer-celebrity relationships among young adults. Hum. Commun. Res..

[54] Belk R. (2013). Extended self in a digital world. J. Consum. Res..

[55] Rasmussen E.E., Riggs R.E., Sauermilch W.S. (2021). Kidfluencer exposure, materialism, and U.S. tweens' purchase of sponsored products. J. Child. Media.

[56] Festinger L. (1954). A theory of social comparison processes. Hum. Relat..

[57] Dinh T.C.T., Lee Y. (2022). “I want to be as trendy as influencers” – how “fear of missing out” leads to buying intention for products endorsed by social media influencers. J. Res. Indian Med..

[58] Yang C.-C. (2016). Instagram use, loneliness, and social comparison orientation: interact and browse on social media, but don't compare. Cyberpsychol., Behav. Soc. Netw..

[59] Pang H. (2021). Unraveling the influence of passive and active WeChat interactions on upward social comparison and negative psychological consequences among university students. Telematics Inf..

[60] Good M., Hyman M. (2021). Direct and indirect effects of fear‐of‐missing‐out appeals on purchase likelihood. J. Consum. Behav..

[61] Kang I., Cui H., Son J. (2019). Conformity consumption behavior and FoMO. Sustainability.

[62] Seo E.J., Park J.W. (2018). A study on the effects of social media marketing activities on brand equity and customer response in the airline industry. J. Air Transport. Manag..

[63] Podoshen J., Andrzejewski S. (2012). An examination of the relationships between materialism, conspicuous consumption, impulse buying, and brand loyalty. J. Market. Theor. Pract..

[64] Field A., Miles J., Field Z. (2012).

[65] Belanche D., Flavián M., Ibáñez-Sánchez S. (2020). Followers' reactions to influencers' Instagram posts. Spanish Journal of Marketing - ESIC.

[66] Ross C., Orr E.S., Sisic M., Arseneault J.M., Simmering M.G., Orr R.R. (2009). Personality and motivations associated with Facebook use. Comput. Hum. Behav..

[67] Solberg E., Diener E., Wirtz D., Lucas R., Oishi S. (2002). Wanting, having, and satisfaction: examining the role of desire discrepancies in satisfaction with income. J. Pers. Soc. Psychol..

[68] Richins M. (2004). The material values scale: a Re-inquiry into its measurement properties and the development of a short form. J. Consum. Res..

[69] O'Cass A., McEwen H. (2004). Exploring consumer status and conspicuous consumption. J. Consum. Behav..

[70] Geiger M., Moore K. (2022). Attracting the crowd in online fundraising: a meta-analysis connecting campaign characteristics to funding outcomes. Comput. Hum. Behav..

[71] Hair J.F., Babin B.J., Anderson R.E., Black W.C. (2018). Multivariate Data Analysis.

[72] Pesämaa O., Zwikael O., Hair J.F., Huemann M. (2021). Publishing quantitative papers with rigor and transparency. Int. J. Proj. Manag..

[73] Hair J., Hult G., Ringle C., Sarstedt M.J.S.P.I. (2022). A Primer on Partial Least Squares Structural Equation Modeling (PLS-SEM).

[74] Hayes A.F. (2018).

[75] Shan Y., Chen K.-J., Lin J.-S. (2020). When social media influencers endorse brands: the effects of self-influencer congruence, parasocial identification, and perceived endorser motive. Int. J. Advert..

[76] Van den Broeck A., Howard J.L., Van Vaerenbergh Y., Leroy H., Gagné M. (2021). Beyond intrinsic and extrinsic motivation: a meta-analysis on self-determination theory's multidimensional conceptualization of work motivation. Organizational Psychology Review.

[77] Demir K. (2011). Teachers' intrinsic and extrinsic motivation as predictors of student engagement: an application of self-determination theory. Educ. Sci..

[78] Moller A.C., Ryan R.M., Deci E.L. (2006). Self-determination theory and public policy: improving the quality of consumer decisions without using coercion. J. Publ. Pol..

[79] Zhang Z., Jiménez F.R., Cicala J.E. (2020). Fear of missing out scale: a self-concept perspective. Psychol. Market..

[80] La Ferle C., Chan K. (2008). Determinants for materialism among adolescents in Singapore, young consumers. Insight and Ideas for Responsible Marketers.

[81] Chan K. (2008). Social comparison, imitation of celebrity models and materialism among Chinese youth. Int. J. Advert..

[82] Fox J., Moreland J.J. (2015). The dark side of social networking sites: an exploration of the relational and psychological stressors associated with Facebook use and affordances. Comput. Hum. Behav..

[83] Zheng X., Baskin E., Peng S. (2018). Feeling inferior, showing off: the effect of nonmaterial social comparisons on conspicuous consumption. J. Bus. Res..

[84] Munnukka J., Uusitalo O., Toivonen H. (2016). Credibility of a peer endorser and advertising effectiveness. J. Consum. Market..

[85] Kim D.Y., Kim H.-Y. (2021). Influencer advertising on social media: the multiple inference model on influencer-product congruence and sponsorship disclosure. J. Bus. Res..

